# Whole-genome association analysis to identify markers associated with recombination rates using single-nucleotide polymorphisms and microsatellites

**DOI:** 10.1186/1471-2156-6-S1-S51

**Published:** 2005-12-30

**Authors:** Song Huang, Shuang Wang, Nianjun Liu, Liang Chen, Cheongeun Oh, Hongyu Zhao

**Affiliations:** 1 Program of Computational Biology and Bioinformatics, Yale University, New Haven, CT 06520, USA; 2 Department of Biostatistics, Mailman School of Public Health, Columbia University, New York, NY 10032, USA; 3 Department of Epidemiology and Public Health, Yale University, New Haven, CT 06520, USA; 4 Department of Biostatistics, University of Alabama at Birmingham, Birmingham, AL 35294, USA; 5 Department of Molecular, Cellular and Developmental Biology, Yale University, New Haven, CT 06520, USA; 6 Division of Biostatistics, Department of Preventive Medicine, University of Medicine and Dentisry of New Jersey, Newark, NJ 07101, USA; 7 Department of Genetics, Yale University, New Haven, CT 06520, USA

## Abstract

Recombination during meiosis is one of the most important biological processes, and the level of recombination rates for a given individual is under genetic control. In this study, we conducted genome-wide association studies to identify chromosomal regions associated with recombination rates. We analyzed genotype data collected on the pedigrees in the Collaborative Study on the Genetics on Alcoholism data provided by Genetic Analysis Workshop 14. A total of 315 microsatellites and 10,081 single-nucleotide polymorphisms from Affymetrix on 22 autosomal chromosomes were used in our association analysis. Genome-wide gender-specific recombination counts for family founders were inferred first and association analysis was performed using multiple linear regressions. We used the positive false discovery rate (pFDR) to account for multiple comparisons in the two genome-wide scans. Eight regions showed some evidence of association with recombination counts based on the single-nucleotide polymorphism analysis after adjusting for multiple comparisons. However, no region was found to be significant using microsatellites.

## Background

Recombination between two homologous chromosomes during meiosis generates novel gene combinations and creates genetic diversity among chromosomes. Furthermore, recombination is critical for proper segregation of homologous chromosomes, and is a major factor shaping linkage disequilibrium (LD) patterns in the genome [1]. Much research has been done recently to establish human genetic maps based on recombination and on estimating local recombination rates to augment LD studies and aid in LD study design and interpretation [1-8]. Kong et al. [2] found marked regional differences in recombination rates and concluded that DNA changes contributing to evolution may not be completely random, but more concentrated within specific regions. This difference may be driven by sequence features. In addition, recombination rate is under genetic control, as exemplified in the finding by Ji et al. [9] that maize meiotic mutant *desynaptic *is a recombination modifier that controls recombination rates. In this study, seeking to identify regions potentially affecting recombination rates, we conducted genome-wide association studies based on microsatellites and single-nucleotide polymorphisms (SNPs) of the Collabroative Study on the Genetics of Alcoholism (COGA) data provided by Genetic Analysis Workshop 14 (GAW14). A total of 315 microsatellites and 10,081 SNPs from Affymetrix on 22 autosomal chromosomes were analyzed. We found eight regions/thirteen SNPs that showed some evidence of association with recombination counts. No region was found to be significant using microsatellites after adjusting for multiple comparisons based on the positive false discovery rate (pFDR) criterion.

## Methods

### Recombination Counts

The COGA data consist of 143 pedigrees with 1,614 individuals, including 1,109 male and female meioses. Genetic maps for microsatellites and SNPs were both provided by GAW14. Some of the distinct SNPs have the same genetic map position, which made inferring recombination events between these SNPs impossible. Therefore, we added 1.0 × 10^-6 ^at these SNPs' genetic map positions to make them distinguishable. To estimate the number of both maternal and paternal recombination events for each female or male meiosis, we used the *Best *option in the haplotyping analysis in MERLIN [10], which outputs the most likely haplotype as well as the most likely sites for recombination throughout a pedigree. The total number of gender-specific recombination counts for each parent was obtained by averaging the numbers of recombination events of all the offspring, which was calculated as the total number of recombination events observed in the 22 autosomal chromosomes. For pedigrees with only two generations, i.e., the nuclear families, the inferred average total number of recombination events from each meiosis of the founders was then treated as a quantitative trait and genome-wide association tests were conducted to identify markers associated with this quantitative trait. For the pedigrees with three or more generations, only recombination information from the founders were extracted and considered in the association tests. We compared the results from the two scans using either microsatellites or SNPs.

### Genotyping error detection

Because genotyping error may lead to double recombinations within a short distance, it can significantly affect the overall recombination counts. To minimize this impact, the error-checking algorithm implemented in MERLIN, which identifies unlikely genotypes based on double recombination events, was applied and the erroneous genotypes were excluded before applying haplotyping analysis. We used the default parameter in MERLIN, where the erroneous genotypes with a likelihood ratio *p *≤ 0.025 were excluded [11]. The same procedure was applied to both SNPs and microsatellies.

### Association analysis to identify markers associated with recombination rates

We used multiple linear regressions to evaluate the relation between recombination counts and markers across 22 autosomal chromosomes with adjustments for age and gender for both SNPs and microsatellites. Analysis was carried out based on Whites only to reduce potential confounding factors related to ethnic differences. To account for the multiple comparison problem in the two whole-genome scans, we used pFDR through *q*-values [12], where a cut-off point of 5% is chosen. The *q*-value is a measure of significance in terms of the pFDR, and it is defined to be the minimum pFDR at which the statistic can be called significant. A pFDR of 5% means that among all of the features that are called significant, 5% of them may correspond to the true null hypotheses on average. To get the *q*-value for each marker, we used the software QVALUE [12] on the *p*-values obtained from the multiple regressions.

## Results and Discussion

### Recombination counts

**Figure 1:**
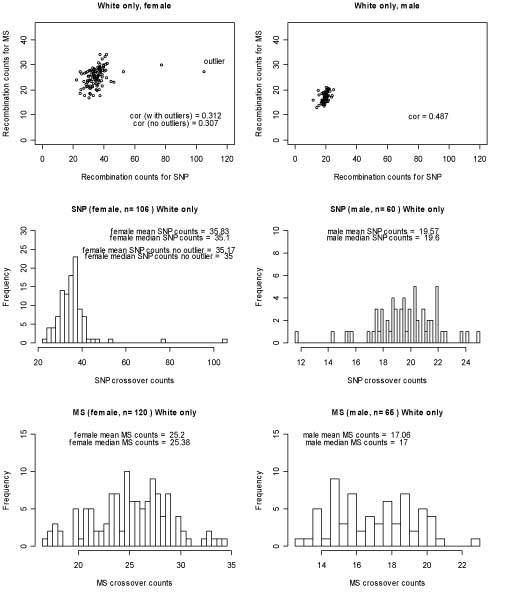
**Distribution of the gender-specific recombination counts**. Results shown are for Whites only when erroneous genotypes are excluded and whenfounders with all genotype information missing are excluded as well. MS, microsatellite.

The genome-wide gender-specific recombination counts of the founders were obtained through averaging recombination counts in all meioses leading to his/her offspring. For SNPs, we inferred the founders' genome-wide recombination counts from the gametes of 1,334 offspring from 130 nuclear families. For microsatellites, we inferred the founders' genome-wide recombination counts from the gametes of 1,409 offspring from 111 nuclear families. This resulted in 189 founders (121 females and 68 males) who were Whites with information for SNPs and 199 founders (129 females and 70 males) who were Whites with information for microsatellies. Among these founders, 14 of them missed all microsatellite genotype information and 23 of them missed all SNP genotype information. They had no contribution in the multiple regression analysis. Therefore, the distributions of the founders' sex-specific recombination counts plotted in Figure 1 did not include these founders. We then had 166 founders (106 females and 60 males) that were Whites with information for SNPs and 185 founders (120 females and 65 males) that were Whites with information for microsatellies. The scatter plots of the inferred recombination counts using SNPs and microsatellites for Whites only showed a higher correlation for males than for female. The recombination counts were much higher for females than for males, a well known biological fact [2,4]. We also noted that the recombination counts were higher using SNPs than those using microsatellites, which may be due to the fact that the SNPs were more dense than the microsatellites, allowing for the capture of recombination events missed by the microsatellites. The mean and median genome-wide gender-specific recombination counts are summarized in Table 1. From the scatter plot for the females, we noted that there were two female founders who had very high inferred recombination counts using the SNPs, 105.4 and 77.8, respectively. In our analysis, we removed the female founder with the average recombination count of 105.4. The above analysis was conducted after removing the possible erroneous genotypes. There were 1,295 microsatellite genotypes that were likely to be erroneous and were set missing with the MERLIN's error checking algorithm, making the estimated genotyping error rate for the microsatellite to be 0.367%. Among the 1,614 individuals and the 315 microsatellites, there were a total of 353,015 genotypes. Similarly, there were 27,338 SNP genotypes that were likely to be erroneous and were set missing with the MERLIN's error checking algorithm. This led to the estimated genotyping error rate for the SNPs to be 0.204% from among the 1,614 individuals and the 10,081 SNPs genotyped. There were a total of 13,395,832 genotypes examined.

We noted that our inferred female and male genome-wide recombination counts were slightly lower than that from previous studies [4]. One reason may be that the 10,081 SNPs did not cover the entire 22 autosomes since the updated SNP data from Affymetrix were not included in the analysis. Another possible reason was that some portion of the corrected genotypes was excluded as erroneous genotypes from the genotyping error detection algorithm.

### Markers associated with recombination counts

Multiple linear regressions with adjustments for age and gender generated *p*-values for each marker, which were not adjusted for multiple comparisons. The corresponding *q*-values based on the pFDR were calculated using the software QVALUE. We applied the 0.05 *q*-value cut-off, which gave us 8 regions/13 SNPs that showed some evidence of association with recombination counts. The positions of those regions together with the raw *p*-values and *q*-values were summarized in Table 2. The 0.05 *q*-value cut-off suggested that 1 out of these 13 SNPs may not be associated with recombination counts. For microsatellites, no region was found to be significant after adjusting for multiple comparisons using pFDR.

## Conclusion

In summary, we have identified several candidate SNPs likely associated with recombination events, and further studies on these genes may help us gain valuable knowledge on recombination, better understand LD patterns, and lead to more efficient methods to map disease genes.

## Abbreviations

COGA: Collaborative Study on the Genetics on Alcoholism

GAW14: Genetic Analysis Workshop 14

LD: Linkage disequilibrium

pFDR: Positive false discovery rate

SNP: Single-nucleotide polymorphism

## Authors' contributions

SH participated in the design of the study, performed the analysis, and drafted the manuscript. SW helped to obtain recombination counts and preparation of the manuscript. SW, NL, LC, and CO participated in the design and the discussion of the study. HZ conceived the study and helped to draft the manuscript. All authors read and approved the final manuscript.

## Figures and Tables

**Table 1:** Mean and median genome-wide gender-specific recombination counts using SNPs and microsatellites.

		SNP		Microsatellite	
			
		Mean (SD)	Median	Mean (SD)	Median
Female	+ outlier	35.83 (9.35)	35.1	25.20 (3.74)	25.38
	- outlier	35.17 (6.42)	35.0		
Male		19.57 (2.34)	19.6	17.06 (2.23)	17.0

Results shown are after removing erroneous genotypes and removing founders that have all genotype information missing for Whites only.

**Table 2:** Significant results (*q*-value < 0.05) for genome-wide association analysis for recombination rates using SNPs.

Chr	Position (cM) (Marker)	Unadjusted *p*-value	*q*-value
1q	129.761 tsc0831812	3.99 × 10^-5^	0.032874
	173.005 tsc1229896	3.64 × 10^-6^	0.005141
	176.25^a ^tsc1687896	3.74 × 10^-5^	0.032874
2q	136.452^a ^tsc0333128	1.01 × 10^-5^	0.012482
	167.814 tsc0045403	2.00 × 10^-8^	0.000198
	167.817 tsc1108827	1.21 × 10^-6^	0.001994
3q	70.968 tsc0753329	4.95 × 10^-5^	0.037662
4q	94.916 tsc0056600	8.00 × 10^-8^	0.000264
8q	103.504 tsc1305199	6.00 × 10^-8^	0.000264
10q	45.654 tsc0615240	2.60 × 10^-7^	0.000514
	74.381 tsc0046577	1.45 × 10^-5^	0.015691
13q	50.514 tsc0616973	2.40 × 10^-7^	0.000514
14q	85.42^a ^tsc1112831	1.59 × 10^-5^	0.015691

No significant results are found with microsatellites using the pFDR 0.05 cutoff

^a^indicates that the marker identified was from the markers that had the same map position
